# Double-Face Meets the Bacterial World: The Opportunistic Pathogen *Stenotrophomonas maltophilia*

**DOI:** 10.3389/fmicb.2017.02190

**Published:** 2017-11-09

**Authors:** Felipe Lira, Gabriele Berg, José L. Martínez

**Affiliations:** ^1^Centro Nacional de Biotecnología, Consejo Superior de Investigaciones Científicas, Madrid, Spain; ^2^Institute of Environmental Biotechnology, Graz University of Technology, Graz, Austria

**Keywords:** *Stenotrophomonas maltophilia*, opportunistic pathogens, comparative genomics, pangenome, core genome, antibiotic resistance

## Abstract

Most studies on bacterial virulence focus on the pathogen itself. However, it is important to recall that the in-host behavior and the virulence of bacterial pathogens constitute a complex situation that depends on both the microorganisms and the infected host. While healthy people (the community) is infected by classical pathogenic microorganisms, able to cope with the anti-infection defenses of the host, in the case of people with basal diseases, debilitated or immunodepressed, the range of pathogens able to cause infection is wider and includes the so-named opportunistic pathogens, which lack the inherent ability to cause disease in healthy hosts and rarely produce infections in the community. Some of the most relevant opportunistic pathogens, as *Stenotrophomonas maltophilia*, have an environmental origin and, in occasions, present interesting biotechnological properties. Consequently, it is important knowing whether *S. maltophilia* isolates recovered from infections constitute a specific phylogenetic branch that has evolved toward acquiring a virulent phenotype as it happens in the case of classical pathogens or rather, any member of this bacterial species is capable of producing infection and its pathogenic behavior is mainly a consequence of the host situation. To address this question, we analyzed a set of environmental and clinical *S. maltophilia* strains. Our results indicate that this opportunistic pathogen presents a large core genome and that the distribution of genes in general, and of known virulence determinants in particular, is similar among environmental and clinical isolates. The majority of genes not belonging to the *S. maltophilia* core genome are present in just one or two of the analyzed strains. This indicates that, more than speciation into different lineages (virulent and environmental), the evolution of *S. maltophilia* is based in the strain-specific acquisition of genes, likely involved in the adaptation of this bacterial species to different microniches. In addition, both environmental and clinical isolates present low susceptibility to several antimicrobials. Altogether our results support that *S. maltophilia* does not present a specific evolutionary branch toward virulence and most likely infection is mainly the consequence of the impaired anti-infective response of the infected patients.

## Introduction

*Stenotrophomonas maltophilia* is an opportunistic pathogen, with an environmental origin, which causes a variety of infections at hospitals (Brooke, 2012; Chang et al., 2015; Jeon et al., 2016; Brooke et al., 2017), particularly in those patients under previous therapy with broad-spectrum antibiotics (Chang et al., 2015), and in patients with underlying diseases as cystic fibrosis (Nicoletti et al., 2011; Pompilio et al., 2016; Esposito et al., 2017). *S. maltophilia* infections are difficult to treat because this pathogen displays low susceptibility to several antimicrobials (Sanchez et al., 2009; Sanchez, 2015). As the consequence of this situation and likely also because *S. maltophilia* infects mainly severely debilitated individuals, the mortality of patients suffering *S. maltophilia* infections is high (Jeon et al., 2016). Consequently, understanding the underlying features by which this pathogen can traverse different ecological allocations, from its natural habitat toward infecting humans, may help in the development of strategies to improve the treatment of infections due to this microorganism.

Besides its clinical relevance, different *S. maltophilia* strains exert an extraordinary range of activities with biotechnological relevance (Mukherjee and Roy, 2016), such as bioremediation (Dungan et al., 2003; Berg and Martinez, 2015), degradation of toxic compounds (Lee et al., 2002), biosynthesis (Jakobi et al., 1996; Nangia et al., 2009; Zonaro et al., 2015) and biological control in agriculture (Dunne et al., 2000; Alavi et al., 2013), among others.

Given these two aspects of *S. maltophilia*, it is highly relevant to determine whether infective and environmental (non-clinical) *S. maltophilia* isolates constitute different evolutionary branches in this species as it has been shown in the case of the *Burkholderia cepacia* complex (Chiarini et al., 2006) or if, by contrary, any strain can infect a compromised human host, as it has been described for *Pseudomonas aeruginosa* (Alonso et al., 1999; Morales et al., 2004; Wiehlmann et al., 2007). This is particularly relevant in order to evaluate the risks for human health associated to the use of *S. maltophilia* for biotechnological purposes, mainly for non-confined applications, as agriculture.

Different works, based in classical Multi-Locus Sequence Typing (MLST), *in silico* MLST and whole genome analyses, have been published to address the phylogenetic structure of this species and of others belonging to the same complex (Rocco et al., 2009; Adamek et al., 2014; Gherardi et al., 2015; Youenou et al., 2015; Esposito et al., 2017; Ochoa-Sanchez and Vinuesa, 2017). Nevertheless, it is still unclear whether or not clinical isolates are predominant in any of these branches. In addition, studies on the potential correlation between the presence in the genome of virulence determinants and antibiotic resistance with the origin of the strains (clinical or environmental) are extremely limited, despite the relevance of these features for the nosocomial infections produced by *S. maltophilia.*

In order to address whether or not clinical and environmental isolates belong to different phylogenetic branches in *S. maltophilia*, in the present work we have sequenced 20 *S. maltophilia* isolates (10 from clinical environments and 10 from environmental samples). Four complete genomes sequences were also included in the study as references, two clinical strains *S. maltophilia* K279a (Crossman et al., 2008) and D457 (Lira et al., 2012) and two environmental isolates, *S. maltophilia* R551-3 (Lucas et al., 2008) and JV3 (Lucas et al., 2011). In addition, in the present work we present the phenotypic analysis of the studied isolates in order to determine whether or not clinical isolates are more resistant to antibiotics than environmental ones, information that cannot be obtained from the simple inspection of *S. maltophilia* available genomes.

## Materials and Methods

### DNA Extraction and Genome Sequencing of 20 New Strains of *Stenotrophomonas maltophilia*

The complete DNAs of 20 isolates of *S. maltophilia* (**Table 1**) were extracted using the GENOME DNA Kit (MP Biomedicals LLC, Illkrich, France). Whole-genome sequencing was performed at the facility of the Madrid Science Park (Madrid, Spain), using Illumina MiSeq technology (Illumina, San Diego, CA, United States) from DNA libraries with insertion sizes between 700 and 800 bp, to generate paired-end reads with 260–300 bp length.

**Table 1:** Accession numbers of the genomes and origins of the *S. maltophilia* isolates of used in this study.

	Strains	Accession number	Origin	Reference
Clinical strains	E729	NERH00000000	Urine	Alonso and Martinez, 2001
	E759	NERG00000000	Sputum	Alonso and Martinez, 2001
	E999	NERF00000000	Respiratory secretion	Alonso and Martinez, 2001
	G51	NERE00000000	Blood	Alonso and Martinez, 2001
	E301	NERD00000000	Urine	Alonso and Martinez, 2001
	D388	NERC00000000	Urine	Alonso and Martinez, 2001
	E861	NERB00000000	Sputum	Alonso and Martinez, 2001
	C357	NERA00000000	Urine	Alonso and Martinez, 2001
	E539	NEQZ00000000	Pus from a wound	Alonso and Martinez, 2001
	E824	NEQY00000000	Blood	Alonso and Martinez, 2001
	K279a^∗^	NC_010943.1	Blood	Crossman et al., 2008
	D457^∗^	NC_017671.1	Respiratory secretion	Lira et al., 2012
Environmental strains	NS26	NEQO00000000	Dune soil	De Boer et al., 2001;Ribbeck-Busch et al., 2005
	EP13	NEQX00000000	Rhizosphere of oilseed rape	Minkwitz and Berg, 2001
	EA22	NEQW00000000	Sewage	Minkwitz and Berg, 2001
	EA1	NEQV00000000	Brackish water	Minkwitz and Berg, 2001
	PS5	NEQU00000000	Rhizosphere of oilseed rape.	Berg et al., 1996
	EA23	NEQT00000000	Eye-care solution	Suckstorff and Berg, 2003
	EP20	NEQS00000000	Rhizosphere of potato	Minkwitz and Berg, 2001
	EP5	NEQR00000000	Rhizosphere of *Brassica napus* L.	Minkwitz and Berg, 2001
	EA21	NEQQ00000000	Sewage	Minkwitz and Berg, 2001
	EA63	NEQP00000000	Sewage	Gabrielle Berg’s lab collection
	R551-3^∗^	NC_011071.1	Endosphere	Lucas et al., 2008
	JV3^∗^	NC_015947.1	Rhizosphere	Lucas et al., 2011


*^∗^Complete genomes of *S. maltophilia.**

### Quality Control and Sequence Assembling

Quality score of the sequences of all strains was checked using FastQC v.0.11.2, to identify adapters and contaminant sequences remaining after sequencing. Contaminant sequences were removed using the AlienTrimmer v.0.4.0 software (Criscuolo and Brisse, 2013) and a customized database of adapters adding the contaminant sequences recognized by FastQC. Sequence trimming and filtering were performed by PRINSEQ-Lite (Schmieder and Edwards, 2011) to filter the sequences by length and quality score (Phred ≥ 22, minimum read length = 90 bp). Each set of reads was submitted to *de novo* assembling using the Spades v.3.9 assembler (Bankevich et al., 2012) in a local server (24 cores and 512Gb RAM). After assembling, contigs with a minimal 5.000 bp length were selected. The synteny of the generated contigs was ordered using Mauve aligner (Darling et al., 2004) and two reference genomes, the model strains *S. maltophilia* D457 (Lira et al., 2012) and *S. maltophilia* K279 (Crossman et al., 2008). Both genomes were chosen because they were the largest complete genomes available. Contigs alignment did not presented divergences with respect to the reference genomes synteny.

### Open Reading Frames Detection, Gene Prediction and Annotation

For the prediction and annotation of the Open Read Frames (ORFs) from each set of contigs, we used two approaches: (a) In a first step the ORFs were predicted using Prodigal v2.6.1 (Hyatt et al., 2010), avoiding truncated genes The parameters were set to predict genes containing both start and stop codons. This approach allowed the elimination of fragmented genes located at the edges of the contigs. Predicted ORFs were annotated performing a local alignment with BLASTp (Camacho et al., 2009) against the NCBI non-redundant database setting the expected value (*e-value*) of 10^-10^. In a second step, all contigs were submitted to the NCBI Prokaryotic Genome Annotation Pipeline (PGAP). Divergences between the local annotation and the PAGP were checked and curated manually.

### Comparative Genomics

Twenty draft genomes of *S. maltophilia* obtained in this study, and the complete genomes of four strains were used to estimate the preliminary core genome and pangenome sizes of *S. maltophilia.* The complete genomes of two clinical strains: D457 (NC_017671.1) and K279a (NC_010943.1) and of two environmental strains: R551-3 (NC_011071.1) and JV3 (NC_015947.1) were also used for the analysis. The accession numbers of draft genomes of the 20 strains of *S. maltophilia* analyzed in this study are: clinical strains: E729 (NERH00000000), E759 (NERG00000000), E999 (NERF00000000), G51 (NERE00000000), E301 (NERD00000000), D388 (NERC00000000), E861 (NERB00000000), C357 (NERA00000000), E539 (NEQZ00000000), E824 (NEQY00000000); environmental strains: NS26 (NEQO00000000), EP13 (NEQX00000000), EA22 (NEQW00000000), EA1 (NEQV00000000), PS5 (NEQU00000000), EA23 (NEQT00000000), EP20 (NEQS00000000), EP5 (NEQR00000000), EA21 (NEQQ00000000), EA63 (NEQP00000000) (**Table 1**).

The pangenome and the core genome of the sequenced strains were analyzed using the script GET_HOMOLOGUES v.07112016 (Contreras-Moreira and Vinuesa, 2013). Clusters of homologous gene families were generated using the COGtriangles algorithm. To form clusters and estimate the core genome and pangenome of *S. maltophilia*, coverage and identity thresholds of 90% and of 95%, respectively were used.

The complete Coding DNA Sequence (CDS) composition and the clusters generated for all strains were used to perform a comparative analysis and to calculate the genome similarity distance to determine the relationship of clinical and environmental isolates. Clustered genes were used to compile the corresponding pangenome matrix using the script compare_cluster.pl with default settings, embedded in the GET_HOMOLOGUES software package. The clusters formed were classified considering the distribution of ortholog genes through the strains. The core genome contains those genes belonging to all strains, the soft-core genome the genes present in, at least, 95% of the strains, the shell genome the genes present in less than 95% and more than 10% of the genomes and the cloud genome the genes present in less than 10% of the genomes (Koonin and Wolf, 2008; Kaas et al., 2012).

### *In Silico* Multi-Locus Sequences Typing and Polymorphic Sites in the Core Genome

*In silico* MLST analysis (Larsen et al., 2012) was performed using the web server of the Centre for Genomic Epidemiology^1^. The alleles from each strain were identified individually and their nucleotides sequences were further concatenated (separated by 10 Ns) to perform a Multiple Sequence Alignment (MSA) using ClustalW2. A phylogenetic tree based in this alignment was calculated using the same software based on the similarity distance between concatenated sequences.

The identification of polymorphic sites was performed using Snippy^2^ using *S. maltophilia* K279a as reference strain (Accession number: NC_010943). Polymorphic sites in genes shared by all strains formed the core of Single Nucleotide Polymorphisms SNPs, that was used to perform a MSA. A phylogenetic tree from the derived information was constructed by using the maximum likelihood method.

### Genomic Composition and Comparative Genomics

Putative functional similarities and differences between the clinical and the environmental strains were estimated by a subsystem classification using the RAST server^3^ (Aziz et al., 2008) and the coding sequences from each genome were classified according to their protein families (*FIGfams*). All strains were compared by the presence/absence of 20 subsystems and 35 functional roles included in the category of “Virulence, Disease and Defense”. A local database containing a set of specific genes, described as responsible for the virulence phenotype of *S. maltophilia* (Adamek et al., 2014) was used to retrieve similar genes from the studied strains. Hierarchical clustering was performed in R functions (Langfelder and Horvath, 2012). For this purpose, each one of the resulting tables containing the information about presence/absence of these genes was converted into a square similarity matrix to measure the distance between strains (R function ‘dist’), clustered based on the matrix data (R function ‘hclust’) and plotted as heatmap (R function ‘heatmap.2’).

### Quorum-Sensing Signals

It has been described that the alleles of the quorum-sensing system (QS) *rpfF* gene, *rpf*F1 (GenBank: KJ149475) and *rpf*F2 (GenBank: KJ149552), are markers of two different phylogenetic branches, each one presenting differences in terms of virulence (Huedo et al., 2014). To address whether or not the presence of a specific *rpfF* allele could be linked to clinical strains, the 108 N-terminal residues of RpfF, which has been proposed to be used as markers for distinguishing the two RpfF variants (Huedo et al., 2014) were aligned using ClustalW2 (Larkin et al., 2007). A phylogenetic tree derived from this information was established using JalView v.2 (Waterhouse et al., 2009)

### Antibiotic Susceptibility

Minimum Inhibitory Concentrations (MICs) were determined in Mueller Hinton agar medium using MIC Test strips (Liofilchem) of the following antibiotics Trimethoprim/Sulfamethoxazole (SXT); Tigecyclin (TGC); Ceftazidime (CAZ); Cefepime (PM); Gentamicin (CN); Gatifloxacin (GAT); Colistin (CS); Chloramphenicol (CL); Imipenem (IMI); Ertapenem (ETP); Moxifloxacin (MXF); Nalidixic Acid (NA).

## Results and Discussion

### Genome Assembling and Annotation of Clinical and Environmental Strains of *Stenotrophomonas maltophilia*

**FIGURE 1:**
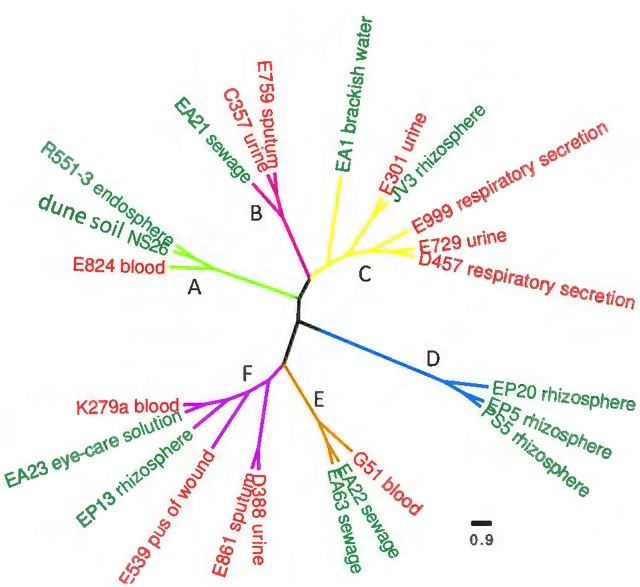
Genetic similarity of clinical and environmental *S. maltophilia* isolates. The complete CDS composition of all strains was used to generate a presence/absence matrix. Based in this matrix, a similarity plot was generated. Red clinical isolates, green environmental strains. As shown, most clusters present both clinical and environmental strains. The horizontal bar shows the genomic similarity distance based on the presence/absence of CDS in each genome.

Although the number of sequenced genomes of the opportunistic pathogen *S. maltophilia* has increased since the first genome was published, specific analyses on the core genome and pangenome (Esposito et al., 2017) as well as on the genomic relationships of clinical and environmental isolates of this species are scarce. In addition, the quality (in terms of number of contigs) of the different available draft genomes is diverse, which makes their comparison difficult in occasions. Finally, clear information on the origin of the isolates (clinical or environmental) is not always available, making the use of these sequences difficult for the purposes of this work. Consequently, to analyze whether clinical and environmental isolates present different genomic features or, by contrary they do not form two different phylogenetic branches, we decided to sequence and analyze twenty isolates of *S. maltophilia* for which the origin has been well established (10 clinical and ten environmental). The assembling of all strains generated a total of 94 Mbp comprising 299 contigs. The genome length average of the sequenced strains was 4.7 Mb and their average GC% content 66.36% (**Table 2**). These data were similar to those of the available *S. maltophilia* complete genomes from strains D457, K279a, R551-3 and JV3, whose genome length and GC% content are, in average, 4.6 Mb and 66.57%, respectively. All contigs were submitted to the Prokaryotes Genome Annotation Pipeline (PGAP) (Tatusova et al., 2014) from NCBI, retrieving an average of 4206 CDS/strain (min = 3879; max = 4540) (**Table 2**). A presence/absence matrix was generated and used for the phylogenetic clustering of the different isolates based in the CDS composition of their genomes. As shown in **Figure 1**, and although branch D comprised just strains isolated from the rhizosphere, the other branches included both clinical and environmental isolates. This fact indicates that, at least in a whole view, there is not a clear differentiation in the CDS composition between the genomes of clinical and environmental *S. maltophilia* isolates.

**Table 2:** Overall characteristics of the genomes analyzed in the current article.

	Strains	Bases	Contigs	Largest contig	GC%	Predicted genes
Clinical strains	K279a^∗^	4,851,126	1	4851126	66.3	4354
	D457^∗^	4,769,156	1	4769156	66.8	4254
	E861	4,658,203	31	653242	66.4	4191
	D388	4,659,986	30	740754	66.4	4190
	E539	4,555,541	18	1731480	66.5	4057
	C357	4,810,581	17	954550	66.2	4310
	E824	5,041,912	14	1834293	65.9	4502
	E729	5,005,550	12	1548184	66.6	4540
	E999	4,414,069	11	1140634	66.7	3879
	G51	4,852,740	8	2066793	66.1	4368
	E301	4,428,328	5	3885998	66.8	3965
	E759	4,546,405	4	2470865	66.5	4083
Environmental strains	R551-3^∗^	4,573,969	1	4573969	66.3	4023
	JV3^∗^	4,544,477	1	4544477	66.9	4040
	EA23	4,752,304	29	642831	66.4	4283
	EP13	4,755,757	27	744273	66.4	4281
	NS26	4,689,165	18	1729723	66.2	4152
	EP20	4,625,290	16	2060034	66.1	4087
	EA1	4,752,176	16	918730	66.6	4234
	EA22	4,759,594	10	1721891	66.2	4265
	EA63	4,885,042	10	1847362	66	4390
	EA21	4,732,256	9	1707011	66.2	4246
	PS5	4,600,476	7	2135136	66.4	4076
	EP5	4,600,182	7	2134905	66.4	4075


*^∗^Complete genomes of *S. maltophilia*.*

### Effect of the Origin of *S. maltophilia* Isolates in Their Pangenome and Core Genome

**FIGURE 2:**
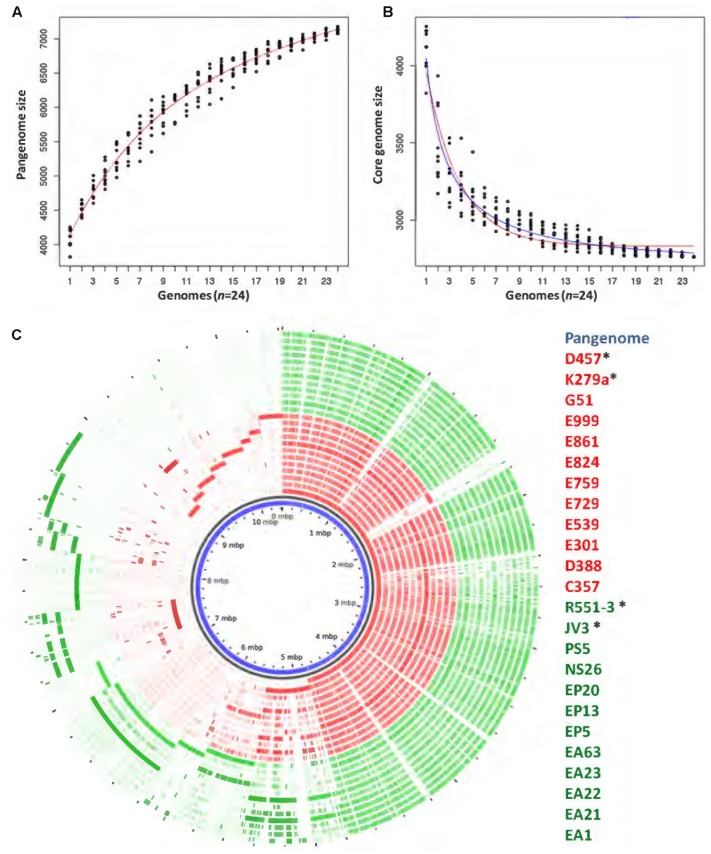
Comparison of the genomes of environmental and clinical *S. maltophilia* isolates. The pangenome and the core genome of *S. maltophilia* were calculated by random sampling of the 24 genomes: **(A)** The number of total genes plotted as a function of the number of genomes added to the analysis is presented. As shown, *S. maltophilia* has an open pangenome. **(B)** The curve shows the number of genes shared by all trains as a function of the number of genomes of *S. maltophilia* added sequentially. Red and blue lines were plotted as an estimation of the tendency of the core genome. Red line indicates that the core genome of *S. maltophilia* should maintain, following the terminology and the estimation rules of Tettelin and collaborators (Tettelin et al., 2005), a ‘relative constancy’ after several genomes are included in the analysis. Blue line indicates that, following the approach of Willenbrock and collaborators, the incorporation of novel genomes might produce a decay in the number of genes that compose the core genome of *S. maltophilia* (Willenbrock et al., 2007). **(C)** Representation of the pangenome obtained by analyzing 24 genomes of *S. maltophilia* isolates. Each circle represents the contribution of each genome to the composition analysis. Genes shared by several strains are clustered at the right side of the circle and strain-specific genes are clustered at its left side. The list of strains displays their names from the inner to the outer circle. ^∗^Complete genomes used in this study. Red: clinical isolates. Green: environmental strains.

**FIGURE 3:**
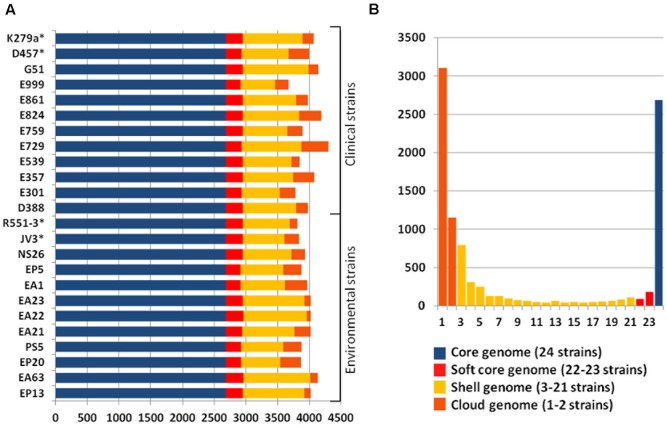
Estimation of *S. maltophilia* core, soft-core, shell and cloud genome. **(A)** Distribution of the predicted CDSs along the 24 *S. maltophilia* genomes representing the core genome (blue bars), the soft-core genome (red bars), the shell genome (yellow bars), and the cloud genome (orange bars) of each of the strains is shown. **(B)** Representation of the overall distribution of genes contributing to the core, the soft, the shell and the cloud genomes. As shown, most genes not belonging to the core/soft-core genome are present in just one or two strains, suggesting that most of the *S. maltophilia* pangenome is strain specific, and does not depend on the environmental or clinical origin of the isolate.

The pangenome and the core genome of *S. maltophilia* were calculated using the draft genomes of the 20 sequenced strains as well as the four full genomes used as references in our work. The number of total genes was plotted as a function of the number of genomes added to the analysis. As shown in **Figure 2A**, an asymptotical increase in the number of genes with respect to the number of analyzed strains was detected. In agreement with previous information (Yu et al., 2016), this indicates that *S. maltophilia* has an open pangenome based on the analysis of the 24 genomes examined. To estimate the core genome, the number of genes shared by all stains was plotted as a function of the number of *S. maltophili*a genomes sequentially added to the analysis (**Figure 2B**). The core genome was estimated in 2762 genes, corresponding to 38% of the pangenome of *S. maltophilia* (**Figure 2B**). To estimate the tendency of the core genome two approaches were performed. Following the approach and terminology of Tettelin et al. (2005), the *S. maltophilia* core genome presents a ‘relative constancy’ after several genomes are included in the analysis (red line in **Figure 2B**), whereas the predictions using the approach of Willenbrock et al. (2007) is that the incorporation of novel genomes should produce a decay in the number of genes that compose the core genome of *S. maltophilia* (blue line in **Figure 2B**). Compositional analysis retrieved a pangenome composed by 7108 orthologous groups, although this number should likely increase when more genomes are analyzed (**Figure 2C**). It is important to notice that, since draft genomes are analyzed, the lack of genes in one specific strain may be the consequence of its presence at the edge of one contig, in which case will be annotated as a truncated gene, although this putative truncation will be the consequence of the method of analysis, not of a real absence. Consequently, the “soft-core genome” (Kaas et al., 2012) was also analyzed. By using this approach, we estimated the number of orthologous genes shared by ∼90% of the organisms included in the comparative analysis. Applying the soft-core genome concept, the number of orthologous clusters increased to 3045. When the 24 genomes were analyzed independently, we estimated that the size of the core genome for each *S. maltophilia* isolate comprised around 59.11% (minimum 54.6%; maximum 64%) of the CDS from each genome. Further, the analysis of the pangenome shows that most of the genes carried by *S. maltophilia* and not belonging to the core genome are strain-specific, suggesting specific adaptations for each isolate more than a common pattern of speciation of some members of the population toward virulence. Indeed, among those genes not belonging to the soft-core genome, and shared by 3–21 strains (dubbed as the ‘shell genome’), just 1337 gene clusters, from the total of 7108 orthologous genes present in the pangenome, were found, indicating that the vast majority of *S. maltophilia* genes, not belonging to its core genome, are strain-specific (**Figure 3**). The speciation of bacterial pathogens usually involve the acquisition by horizontal gene transfer (HGT) of virulence genes, followed by the loss of other genes and the selection of mutations that allow the fine tuning of the metabolism (Martinez, 2013), a process very well studied in the case of *Yersinia* (Achtman et al., 1999; Wren, 2003; Achtman et al., 2004; Zhou and Yang, 2009). HGT is the consequence of either transformation or either the acquisition of mobile elements. Once these mobile elements are acquired, they can be fixed or spread to other hosts, a situation highly relevant in the case of antibiotic resistance (Martinez et al., 2009, 2017). Despite that the presence of several genes in the cloud genome of *S. maltophilia* suggests that this process has largely contributed to the diversification of this pathogen, clear information on its mobilome has not been published. Indeed, only three whole sequenced *S. maltophilia* plasmids are available, which makes difficult to estimate the role of these mobile elements in the evolution of *S. maltophilia*.

In addition to the finding that there are several strain-specific genes, is important to recall that, as **Figure 2C** shows, a differential distribution of genes, not belonging to the core genome, was not found when environmental and clinical isolates of *S. maltophilia* were compared. This result further suggests that there is not a specific phylogenetic branch, deriving from the acquisition of a specific set of virulence determinants by the clinical *S. maltophilia* isolates, which can drive the speciation of this microorganism toward virulence.

It is important to highlight that several articles analyzing bacterial core genomes make use of draft genomes in which genes at the edges of contigs are interrupted, which introduce some noise in the analysis that can produce an underestimation of the size of core genomes. Hence to avoid such noise, and since generation of complete genomes is by far more expensive than draft genomes, we propose using the soft-core genome as the right estimator of the number of genes that are common to all members of a given bacterial species.

### *In Silico* Multi-Locus Sequences Typing (MSLT) and Core Genome SNPs

**FIGURE 4:**
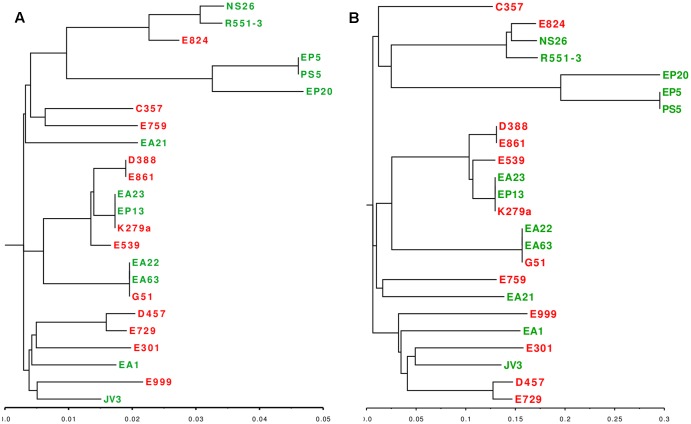
Phylogenetic distribution of clinical and *environmental S. maltophilia* isolates. **(A)** Phylogenetic dendrogram based on the *in* vitro MLST analysis of seven concatenated genes (*atpD, guaA, nuoD, recA, gapA, mutM, ppsA*) **(B)** Phylogenetic dendrogram based on the alignment of SNPs present in the core genome of *S. maltophilia.* Red: clinical isolates. Green: environmental strains. As shown, both analysis grouped the strains in three major clusters, each one containing clinical and environmental strains.

Phylogenetic branches do not depend just on the presence/absence of genes, but in the fixation of specific mutations that can also provide differentiation of clinical and environmental isolates in different phylogenetic branches. To address this possibility, we performed two types of complementary analysis, namely *in silico* MLST and study of the core genome SNPs. Seven genes were used as markers for the MLST analysis: *atpD*, *gapA*, *guaA*, *mutM*, *nuoD*, *ppsA* and *recA*. The phylogenetic tree based on the alignment of these genes consisted of three major groups, each one of them comprised by clinical and environmental strains (**Figure 4A**). All SNPs were identified using *S. maltophilia* K279a as reference and phylogenetic dendrogram based on the core SPNs alignment was consistent with the topology and branches of the MSLT-based tree (**Figure 4B**). The data combining the genotypic profiling provided by the MSLT and the evaluation of the core SNPs of the 24 strains presented in this study revealed that *S. maltophilia* is a diverse complex, forming an interlaced taxon, sharing the same attributes between clinical and environmental strains without preference with respect to their origin.

### Functional-Based Comparison between Clinical and Environmental Strains of *S. maltophilia*

**FIGURE 5:**
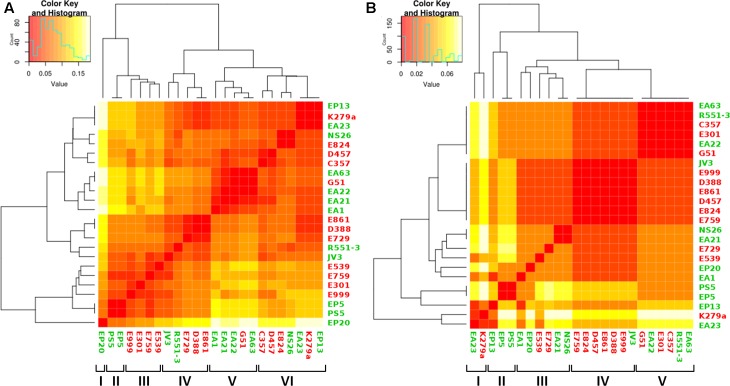
Analysis of genome composition of clinical and environmental *S. maltophilia* isolates based on functional categories. Color key represents the scale of similarities from red (high) to yellow (low), and counts axis is the number of observed pairs (x, y) that fall into each binary event (presence/absence of shared functional categories for each isolate) represented by the histograms (blue lines). Green, environmental isolates; red clinical isolates. **(A)** Heatmap showing the clustering of clinical and environmental *S. maltophilia* isolates based in the genes with functional roles classified at FIGfam within the subsystem ‘Virulence, Disease and Defense’ in the RAST server. The clustering, based on a presence/absence matrix, revealed that most clusters contain both clinical and environmental strains. **(B)** Heatmap showing the clustering of clinical and environmental *S. maltophilia* strains based in the presence/absence of a specific set of virulence determinants described in Adamek et al. (2014). As shown, most branches contain both environmental and clinical isolates.

Even though we did not find a clear distinction between the genome sizes and their CDS composition of clinical and environmental *S. maltophilia* strains, it is still possible that some functional categories, particularly those dealing with virulence are enriched as a function of the habitat (clinical or environmental) from which these strains have been isolated. Consequently, the 20 sequenced genomes and the four complete genomes used as reference were analyzed according to the functional groups of the CDS present in each of the genomes to further explore the relationship between habitat and genome composition. The presence of genes classified into the FIGfam subsystem ‘Virulence, Disease and Defense’ (Overbeek et al., 2005) was analyzed in all strains (Supplementary Table S1). From this information, a customized set of genes, containing only genes that were not present in all the isolates (Supplementary Table S2) was used to create a presence/absence matrix with roles not shared by all strains. Calculation of the average distance of strains and further clustering indicated the formation of six hierarchical clusters (**Figure 5A**). From the six clusters, only clusters II and III were formed exclusively by strains isolated from the same habitat. Environmental strains isolated from the rhizosphere, EP5 and PS5, composed the cluster II and both lacked some functional roles attributed to copper resistance. Cluster III was constituted by the clinical isolates E999, E301, E759 and E539 isolated from respiratory secretion, urine, sputum and pus, respectively. These isolates did not present five functional roles responsible for copper resistance. Other three clusters presented both clinical and environmental isolates in their composition, and EP20 did not group with other strains. Cluster IV, despite its composition including clinical and environmental isolates, presented a subdivision in those branches, creating two distinct sub-clusters composed by two environmental strains isolated from plants, JV3 and R551-3. Clinical isolates E861 and E729 were obtained from patients presenting urinary infection and strain D388 obtained from blood sample. The isolates grouped in cluster V present different origins and were characterized by the presence of genes related with Hg resistance (Supplementary Table S2). The presence of these genes suggest that these strains are able to inactivate Hg toxic forms into less toxic compounds. Finally, cluster VI was characterized by the diversity of organisms isolates from different habitats.

Taking into consideration that most clusters contain clinical and environmental isolates, and that the observed differences do not involve the presence of specific virulence genes in the clinical isolates, our results reinforce the notion that there are not clear distinctions between clinical and environmental *S. maltophilia* strains, even when the analysis is based in the distribution of functional categories.

It is worth mentioning, however, that virulence can be due to the presence of a small subset of genes and global analysis would not be sufficient to distinguish the presence or absence of such genes. Consequently, we screened for the presence of a set of genes that has been described as markers for *S. maltophilia* virulence (Adamek et al., 2014) (Supplementary Table S3). By using this dataset, the presence of five clusters was shown with four of them mixing clinical and environmental strains from different origins (**Figure 5B**). Only cluster II contained exclusively environmental strains (three), all obtained from plant rhizosphere. These results reinforced the idea that the genomic composition is not sufficient to establish a clear separation between clinical and environmental strains of *S. maltophilia*. Cluster I grouped the isolates EA23 and K279a that presents genes encoding filamentous hemaglutinins, which are important for adhesion and spread of bacteria through the respiratory tract (Colombi et al., 2006;Crossman et al., 2008). Despite it was not grouped in the same cluster, the isolate EP13 presented as well filamentous hemaglutinins genes. Isolates EP5 and PS5 were clustered in the same branch, in agreement with their complete CDS composition. Seven isolates did not present five functional roles responsible for copper resistance: four of them, E759, E999, E301 and E539, were clinical strains that shared the same cluster when the analysis was performed using the classification based in functional roles (**Figure 5A**). Otherwise, they did not share the same cluster when analyzed using the set of virulence factors (**Figure 5B**). The same happened with the environmental isolates PS5 and EP5 that shared the same clusters in both types of analysis. Altogether, the phylogenetical relationship of all strains analyzed in this study, calculated in base of their CDS composition and the clustering in orthologous groups, demonstrated that clinical and environmental strains did not form two independent evolutionary lineages. These results support the idea that clinical and environmental isolates are closely related and the pathogenic behavior does not depend on the acquisition of a specific set of virulence genes.

### Quorum-Sensing Signals

The quorum-sensing system (QS) is responsible for the synchronization of particular bacterial behaviors on a population scale. In the case of *S. maltophilia* this process is relevant for *S. maltophilia* virulence and for its interaction with plants (Alavi et al., 2013, 2014), and depends on the Diffusible Signal Factor QS (DSF-QS), which has been identified as the fatty acid *cis*-11-methyl-2-dodecenoic acid (Fouhy et al., 2007).

**FIGURE 6:**
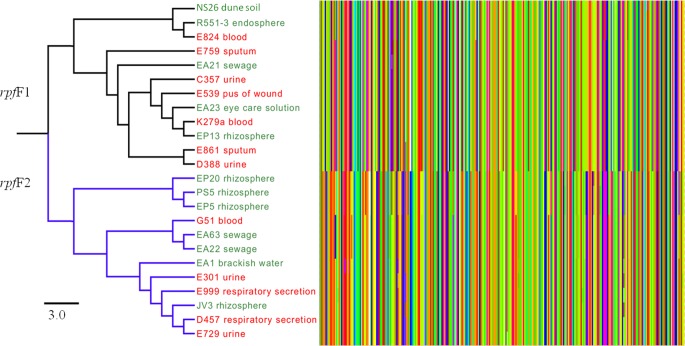
Comparative analysis of the amino acid sequences of the two variants of RpfF of clinical and environmental *S. maltophilia* strains. The Figure shows the alignment of the 108 N-terminal residues of RpfF, which has been proposed to be used as markers for distinguishing the two RpfF variants (Huedo et al., 2014). Each amino acid is represented by a bar with different color. As shown, a clear separation between the two variants of RpfF are detected, but there is not a clear distinction between clinical and environmental isolates. Clinical strains are represented in red and environmental strains in green. The bar shows the sequence similarity distance.

In a previous study the existence of two different alleles for the *rpfF* gene, that is essential for the synthesis of DSF has been described. Each of the alleles defined a branch presenting a different virulence behavior (Huedo et al., 2014). It is then still possible that environmental and clinical isolates could present a differential virulence based in the presence/absence of a specific *rpfF* allele. Since these variants are markers of two different phylogenetic branches, each one presenting differences in terms of virulence, we analyzed their presence in the 24 studied genomes. Using the available sequences for *rpf*F1 and *rpf*F2, a direct search was performed for the corresponding DNA region of *rpf*F in all 24 genomes. All 24 strains of *S. maltophilia* harbor this gene with different length and variable residues along the sequence. In agreement with previous results (Huedo et al., 2014), the studied isolates are distributed into two distinct groups, each one presenting a different *rpf*F variant. Each group comprised 12 strains (**Figure 6**); however, there was not a clear difference in the distribution of clinical and environmental isolates between both groups. The cluster with the RpfF1 variant, which displays detectable DSF production (Huedo et al., 2014), comprised four environmental strains and eight clinical isolates, while the cluster containing the RpfF2 allele, with no significant effect on virulence-related phenotypes, presented seven environmental and five clinical strains.

### Antibiotic Susceptibility of Clinical and Environmental Isolates of *S. maltophilia*

**FIGURE 7:**
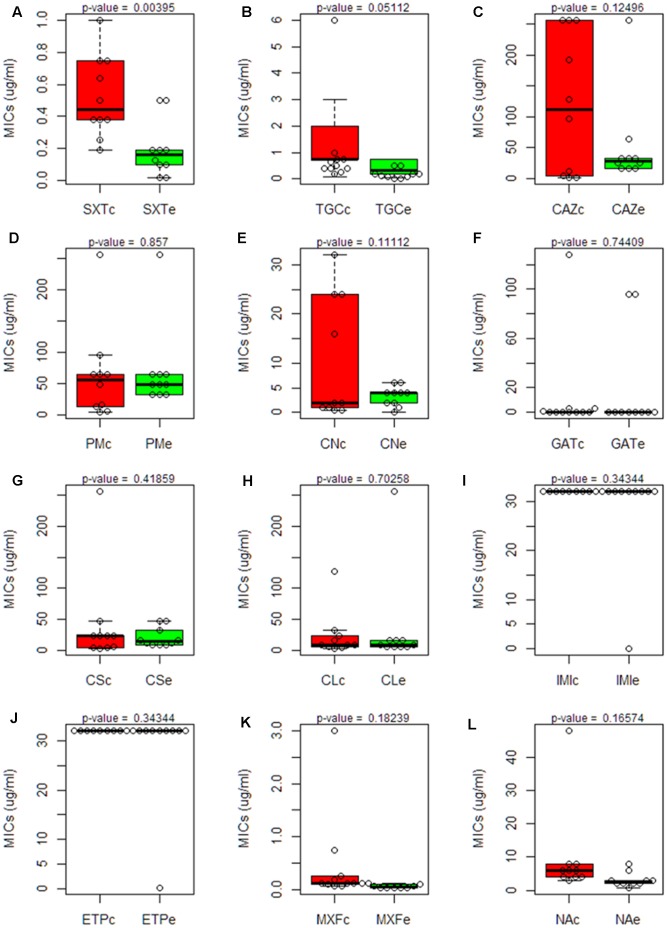
Comparison of the susceptibility to antibiotics of clinical and environmental *S. maltophilia* isolates. Boxplot charts representing the Minimal Inhibitory Concentrations (MICs) for all clinical and environmental isolates obtained using antibiogram strip-tests of 12 antibiotics from different families: (SXT, Trimethoprim/Sulfamethoxazole; TGC, Tigecyclin; CAZ, Ceftazidime; PM, Cefepime; CN, Gentamicin; GAT, Gatifloxacin; CS, Colistin; CL, Chloramphenicol; IMI, Imipenem; ETP, Ertapenem; MXF, Moxifloxacin; NA, Nalidixic Acid). The median and the quartiles for the MIC values in each group are shown. Clinical isolates are represented in red box plots; environmental isolates are represented in green box plots. Statistical significance of the results was estimated by using the *t*-Student test. A significant difference (*p*-value < 0.05) was found just in the case of Trimethoprim/Sulfamethoxazole. Trimethoprim/Sulfamethoxazole (SXT); Tigecyclin (TGC); Ceftazidime (CAZ); Cefepime (PM); Gentamicin (CN); Gatifloxacin (GAT); Colistin (CS); Chloramphenicol (CL); Imipenem (IMI); Ertapenem (ETP); Moxifloxacin (MXF); Nalidixic Acid (NA). Each panel, from **(A–L)** represents the MICs of one antibiotic.

The Minimal Inhibitory Concentrations (MIC) of 12 different antibiotics, belonging to a wide range of structural families and presenting different targets, were established for the 20 isolates. The strain D457, which has been used in several studies on antibiotic resistance in *S. maltophilia* (Alonso and Martinez, 1997) was included as a control. The results (**Table 3**) were plotted in quartiles. As shown in **Figure 7**, the clinical isolates, as a group, present a trend toward higher levels of resistance than the environmental ones.

**Table 3:** Minimal Inhibitory Concentrations (MICs) of 20 new sequenced strains and of the model strain *S. maltophilia* D457.

	Strains	SXT	TGC	CAZ^∗^	PM^∗^	CN	GAT	CS	CL^∗^	IMI^∗^	ETP^∗^	MXF	NA
Clinical strains	E729	0.75	3	1	4	2	1	4	24	>32	>32	0.75	8
	E759	1	2	>256	>256	1	128	6	32	>32	>32	0.25	4
	E999	0.38	2	4	12	24	0.25	4	16	>32	>32	0.13	8
	G51	0.38	0.75	>256	48	2	0.06	24	3	>32	>32	0.09	6
	E301	0.19	0.75	1.5	6	0.38	0.13	24	4	>32	>32	0.19	6
	D388	0.25	0.75	96	64	24	0.13	24	6	>32	>32	0.06	3
	E861	0.38	0.5	128	64	16	0.13	24	8	>32	>32	0.13	4
	C357	0.75	6	192	96	32	3	48	128	>32	>32	3	48
	E539	0.5	0.75	256	64	1	0.13	256	8	>32	>32	0.13	6
	E824	0.64	0.09	12	16	0.5	0.05	3	8	>32	>32	0.06	4
Environmental strains	NS26	0.19	0.75	16	64	2	0.13	12	6	>32	>32	0.13	3
	EP13	0.09	0.19	16	32	4	96	12	16	>32	>32	0.13	0.8
	EA22	0.5	0.75	32	48	2	0.13	32	6	>32	>32	0.03	3
	EA1	0.5	0.19	32	48	4	0.02	8	16	>32	>32	0.03	2
	PS5	0.02	0.05	64	>256	0.09	0.02	16	>256	0.06	0.2	0.03	8
	EA23	0.19	0.25	24	64	4	0.19	8	6	>32	>32	0.09	2
	EP20	0.09	0.19	16	32	6	0.09	48	8	>32	>32	0.03	2
	EP5	0.02	0.75	24	32	4	96	8	6	>32	>32	0.03	2
	EA21	0.19	0.5	>256	64	1	0.09	16	16	>32	>32	0.06	3
	EA63	0.13	0.38	32	48	6	0.06	48	8	>32	>32	0.06	6
	D457	0.13	1.5	1.5	16	6	0.5	32	12	32	32	0.25	8
	MIC50	0.25	0.75	32	48	2	0.13	16	8	32	32	0.09	4
	MIC90	0.75	2	256	96	24	96	48	32	32	32	0.25	8


*Values of the MIC50 (Minimum Inhibitory Concentration required to inhibit the growth of 50% of the isolates) and MIC90 (Minimum Inhibitory Concentration required to inhibit the growth of 90% of the isolates). Antibiotics: SXT, Trimethoprim/Sulfamethoxazole; TGC, Tigecyclin; CAZ, Ceftazidime; PM, Cefepime; CN, Gentamicin; GAT, Gatifloxacin; CS, Colistin; CL, Chloramphenicol; IM, Imipenem; ETP, Ertapenem; MXF, Moxifloxacin; NA, Nalidixic Acid. ^∗^At least one strain presented a MIC above the highest antibiotic concentration in the strip test.*

The environmental strain PS5 was the only isolate susceptible to IMI and ETP, while the other strains grew over the maximum value of this strip-test (>32 μg/ml), a feature that fits with previous information showing that *S. maltophilia* is resistant to these antibiotics (Howe et al., 1997). Notably, the same isolate, PS5, presented the highest level of resistance to CL (>256 μg/ml), followed by the strain C357 (MIC 128 μg/ml). For the other strains, the values ranged between 3 and 32 μg/ml. This may suggest that all *S. maltophilia* isolates, independently from their origin, present similar chances to acquire resistance to this antibiotic.

Although all isolates displayed low susceptibility to the tested antibiotics, when we analyze just the antibiotic concentration ranges where the values did not exceed the maximum concentration of the strip tests, the clinical strains presented overall higher MIC values for the antibiotics SXT, TGC, GAT, MXF and NA when compared with the environmental isolates (**Figure 7**). Nevertheless, this difference was statistically significant only in the case of STX. Therefore, despite there seems to be a trend toward lower MIC values in the environmental isolates, and in agreement with other studies (Berg et al., 1999), the multiple-antibiotic-resistance pattern of both clinical and environmental strains does not present significant differences and might be explained by the intrinsic resistome linked to the core genome of this species.

**FIGURE 8:**
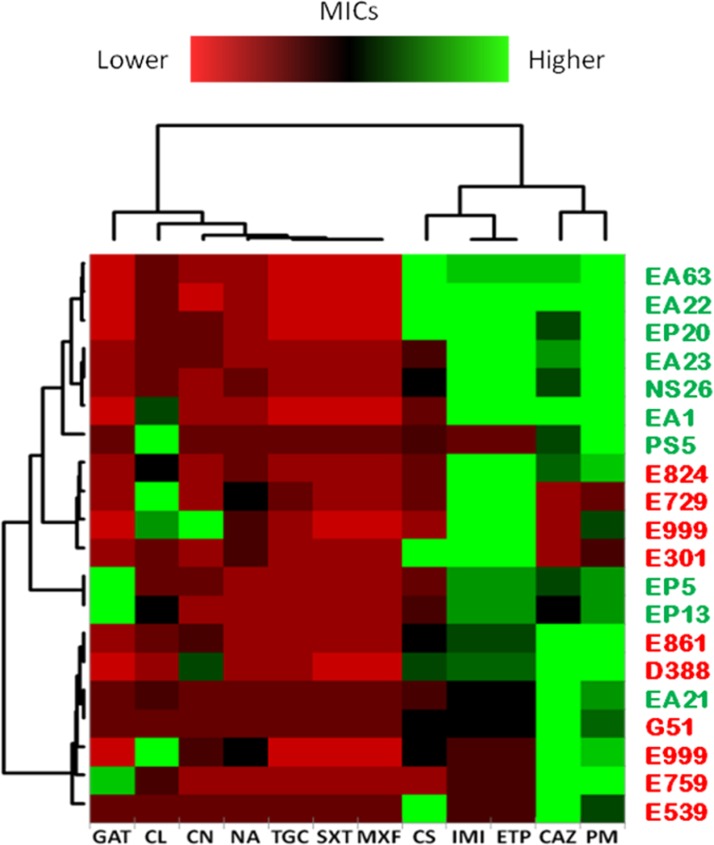
Normalized susceptibility to antibiotics of clinical and environmental *S. maltophilia* isolates. Clustering of strains was obtained by normalizing the MICs from each independent isolate to the MIC50 (minimal concentration at which 50 % of the strains are susceptible) of all strains, and expressed in logarithmic scale (log_2_). Trimethoprim/Sulfamethoxazole (SXT); Tigecyclin (TGC); Ceftazidime (CAZ); Cefepime (PM); Gentamicin (CN); Gatifloxacin (GAT); Colistin (CS); Chloramphenicol (CL); Imipenem (IMI); Ertapenem (ETP); Moxifloxacin (MXF); Nalidixic Acid (NA). Green plots represent MICs higher than MIC50 while red plots represent MICs lower than MIC50. As shown, there is not a common trend toward multi-resistance among clinical isolates when compared with environmental strains.

The previous analysis was based in the independent analysis of each of the antibiotics in the full population. To analyze a different aspect of the problem: the susceptibility to several antibiotics in each independent isolate, further comparisons of the clinical and environmental isolates were performed normalizing the obtained MICs by the MIC50 of all strains (**Figure 8**). Normalization of the MICs by the MIC50 of the 20 isolates grouped the environmental strains NS26, EA22, EA1, PS5, EA23, EP20 and EA63 in one branch presenting overall less susceptibility to carbapenems, imipenem and ertapenem, and the cefalosporins, ceftazidime and cefepime, than the other strains, indicating that, at least for some antibiotics, environmental isolates can present higher levels of resistance than clinical strains. Previous publications have shown that both clinical and environmental *S. maltophilia* isolates are highly resistant to antibiotics (Berg et al., 1999). Our results confirm this issue: the MICs of most antibiotics are high in all isolates as compared with other bacterial pathogens, and in occasions environmental strains are even less susceptible than clinical isolates.

## Conclusion

When looking to the structure of bacterial species presenting infective and non-infective habitats, three situations can be foreseen. Either the species present specific virulence branches, as it happens in the case of *Escherichia coli*, either all isolates can produce an infection in healthy and sick people as in the case of *Yersinia pestis*, either all isolates can produce infection, but only in people with a previous basal disease, as it has been described in the case of *P. aeruginosa*. The consequences in terms of preventing infections by each one of these species would be different. For the first type of microorganisms, surveillance must be taken at the clonal level: some clones constitute a risk while some others are not dangerous. For the second, each member of the species must be considered as a risk for human health. In the third case, the risk is not mainly due to the organism itself, which does not infect the community, but to the situation of the potential host to be infected. Our results indicate that *S. maltophilia* belongs to the third category; all strains are likely equivalent in their capability of infecting humans, but only patients presenting severe underlying diseases including cystic fibrosis would be infected by this pathogen. Given the high biotechnological potential of *S. maltophilia*, both for confined and non-confined applications, there are concerns on the risk that this use may have for human health. Our results indicate that this concern applies just for people with underlying diseases and not for the community and, given that *S. maltophilia* is an environmental ubiquitous and cosmopolitan organism, its use in the habitats that this bacterium regularly colonizes will likely produce just an incremental risk of acquiring infections, even in the case of patients presenting underlying diseases.

A final reflection concerns the distribution of *S. maltophilia* pangenome. Most genes not belonging to the core genome are present in just one or a few strains. Together with the finding that *S. maltophilia* presents an open genome, this suggests that *S. maltophilia* can likely colonize a full range of microniches and, for such colonization, each member of this bacterial species is capable of acquiring a specific set of genes through HGT.

## Author Contributions

FL performed the experiments and bioinformatic analysis presented in the work. All authors contributed to the design and interpretation of the results, as well as to writing the article, and approved it for publication.

## Conflict of Interest Statement

The authors declare that the research was conducted in the absence of any commercial or financial relationships that could be construed as a potential conflict of interest.

## Footnotes

1 www.cbs.dtu.dk/services/MLST

2 https://github.com/tseemann/snippy

3 http://rast.nmpdr.org/
