# Supplementary material for: Double-Face Meets the Bacterial World: The Opportunistic Pathogen Stenotrophomonas maltophilia
Source: Front Microbiol. 2017 Nov 9;8:2190. doi: 10.3389/fmicb.2017.02190 (PMC5684188; doi:10.3389/fmicb.2017.02190)
Supplement: Supplementary file 1 [file Table_1.DOCX]

Supplementary material

Table S1: Number of genes in subsystems detected in the 24 genomes of *S. maltophilia* presented in this study. Genomes of all strains had their genes classified in FIGfams using the RAST server and the amount of genes classified accumulated into each of the subsystems.

|  | **Clinical strains** | | | | | | | | | | | | **Environmental strains** | | | | | | | | | | | |
| --- | --- | --- | --- | --- | --- | --- | --- | --- | --- | --- | --- | --- | --- | --- | --- | --- | --- | --- | --- | --- | --- | --- | --- | --- |
| **Subsystems** | **K279*** | **D457*** | **E729** | **E759** | **E999** | **G51** | **E301** | **D388** | **E861** | **C357** | **E539** | **E824** | **R551-3*** | **JV3*** | **NS26** | **EP13** | **EA22** | **EA1** | **PS5** | **EA23** | **EP5** | **EA21** | **EA63** | **EP20** |
| Tolerance to colicin E2 | 4 | 4 | 4 | 4 | 3 | 3 | 3 | 4 | 4 | 4 | 4 | 4 | 4 | 4 | 4 | 0 | 3 | 3 | 4 | 4 | 4 | 3 | 3 | 0 |
| Colicin V and Bacteriocin Production Cluster | 8 | 8 | 8 | 8 | 8 | 8 | 8 | 8 | 8 | 8 | 8 | 8 | 8 | 8 | 8 | 4 | 8 | 8 | 8 | 8 | 8 | 8 | 8 | 8 |
| Mycobacterium virulence operon involved in protein synthesis (SSU ribosomal proteins) | 4 | 4 | 4 | 4 | 4 | 4 | 4 | 4 | 4 | 4 | 4 | 4 | 4 | 4 | 4 | 8 | 4 | 4 | 4 | 4 | 4 | 4 | 4 | 4 |
| Mycobacterium virulence operon involved in DNA transcription | 2 | 2 | 2 | 2 | 2 | 2 | 2 | 2 | 2 | 2 | 2 | 2 | 2 | 2 | 2 | 4 | 2 | 2 | 2 | 2 | 2 | 2 | 2 | 2 |
| Mycobacterium virulence operon involved in protein synthesis (LSU ribosomal proteins) | 3 | 3 | 3 | 3 | 3 | 3 | 3 | 3 | 3 | 3 | 3 | 3 | 3 | 3 | 3 | 2 | 3 | 3 | 3 | 3 | 3 | 3 | 3 | 3 |
| Listeria surface proteins: Internalin-like proteins | 0 | 0 | 0 | 0 | 0 | 0 | 0 | 0 | 0 | 0 | 0 | 0 | 0 | 0 | 0 | 0 | 0 | 0 | 1 | 0 | 1 | 0 | 0 | 0 |
| MexC-MexD-OprJ Multidrug Efflux System | 1 | 0 | 0 | 1 | 0 | 1 | 0 | 1 | 1 | 1 | 1 | 1 | 1 | 1 | 1 | 3 | 1 | 1 | 0 | 1 | 0 | 1 | 1 | 0 |
| Copper homeostasis | 12 | 12 | 13 | 8 | 8 | 13 | 7 | 12 | 12 | 11 | 8 | 10 | 10 | 11 | 10 | 1 | 13 | 12 | 8 | 12 | 8 | 13 | 13 | 8 |
| Bile hydrolysis | 1 | 1 | 1 | 1 | 1 | 1 | 1 | 1 | 1 | 1 | 1 | 1 | 1 | 1 | 1 | 12 | 1 | 1 | 1 | 1 | 1 | 1 | 1 | 1 |
| Cobalt-zinc-cadmium resistance | 11 | 12 | 12 | 9 | 10 | 11 | 9 | 11 | 11 | 11 | 10 | 11 | 9 | 11 | 11 | 1 | 11 | 12 | 7 | 11 | 7 | 12 | 11 | 7 |
| MDR, Tripartite Systems Found in Gram Negative Bacteria | 3 | 3 | 3 | 3 | 3 | 3 | 3 | 3 | 3 | 3 | 3 | 3 | 3 | 3 | 3 | 11 | 3 | 3 | 3 | 3 | 3 | 3 | 3 | 3 |
| Mercuric reductase | 1 | 1 | 0 | 0 | 0 | 1 | 0 | 0 | 0 | 1 | 0 | 1 | 0 | 0 | 1 | 3 | 1 | 1 | 0 | 1 | 0 | 1 | 1 | 0 |
| Mercury resistance operon | 4 | 4 | 0 | 0 | 0 | 8 | 0 | 0 | 0 | 4 | 0 | 5 | 0 | 0 | 5 | 1 | 7 | 6 | 0 | 4 | 0 | 6 | 8 | 0 |
| Aminoglycoside adenylyltransferases | 1 | 1 | 1 | 1 | 1 | 1 | 1 | 1 | 1 | 1 | 0 | 1 | 1 | 1 | 1 | 4 | 1 | 1 | 1 | 1 | 1 | 1 | 1 | 1 |
| Resistance to fluoroquinolones | 4 | 4 | 4 | 4 | 4 | 4 | 4 | 4 | 4 | 4 | 4 | 4 | 4 | 4 | 4 | 1 | 4 | 4 | 4 | 4 | 4 | 4 | 4 | 0 |
| Arsenic resistance | 5 | 5 | 5 | 5 | 5 | 5 | 4 | 5 | 5 | 5 | 5 | 5 | 5 | 5 | 5 | 4 | 5 | 4 | 5 | 5 | 5 | 5 | 5 | 5 |
| Copper homeostasis: copper tolerance | 3 | 2 | 2 | 2 | 2 | 2 | 3 | 2 | 2 | 2 | 3 | 2 | 3 | 2 | 3 | 5 | 2 | 3 | 2 | 3 | 2 | 2 | 2 | 2 |
| Beta-lactamase | 4 | 4 | 4 | 4 | 4 | 4 | 4 | 4 | 4 | 4 | 4 | 4 | 4 | 4 | 4 | 3 | 4 | 4 | 4 | 4 | 4 | 4 | 4 | 4 |
| Multidrug Resistance Efflux Pumps | 11 | 11 | 10 | 13 | 11 | 12 | 11 | 11 | 11 | 12 | 11 | 12 | 12 | 12 | 12 | 4 | 12 | 12 | 11 | 11 | 11 | 12 | 12 | 11 |
| Resistance to chromium compounds | 0 | 1 | 1 | 0 | 1 | 1 | 0 | 0 | 0 | 1 | 0 | 0 | 1 | 0 | 0 | 11 | 1 | 0 | 0 | 0 | 0 | 1 | 1 | 0 |

Table S2: Simplified matrix of presence/absence of functional roles distribution that differed between strains*.

| **Functional roles** | **K279a#** | **D457#** | **E729** | **E759** | **E999** | **G51** | **E301** | **D388** | **E861** | **C357** | **E539** | **E824** | **JV3#** | **R551-3#** | **NS26** | **EP13** | **EA22** | **EA1** | **PS5** | **EA23** | **EP5** | **EA21** | **EA63** | **EP20** |
| --- | --- | --- | --- | --- | --- | --- | --- | --- | --- | --- | --- | --- | --- | --- | --- | --- | --- | --- | --- | --- | --- | --- | --- | --- |
| Arsenic efflux pump protein |  |  |  |  |  |  |  |  |  |  |  |  |  |  |  |  |  |  |  |  |  |  |  |  |
| Cd(II)/Pb(II)-responsive transcriptional regulator |  |  |  |  |  |  |  |  |  |  |  |  |  |  |  |  |  |  |  |  |  |  |  |  |
| Chromate transport protein ChrA |  |  |  |  |  |  |  |  |  |  |  |  |  |  |  |  |  |  |  |  |  |  |  |  |
| Cobalt-zinc-cadmium resistance protein CzcD |  |  |  |  |  |  |  |  |  |  |  |  |  |  |  |  |  |  |  |  |  |  |  |  |
| Colicin E2 tolerance protein CbrC-like protein |  |  |  |  |  |  |  |  |  |  |  |  |  |  |  |  |  |  |  |  |  |  |  |  |
| CopG protein |  |  |  |  |  |  |  |  |  |  |  |  |  |  |  |  |  |  |  |  |  |  |  |  |
| Copper resistance protein C precursor |  |  |  |  |  |  |  |  |  |  |  |  |  |  |  |  |  |  |  |  |  |  |  |  |
| Copper resistance protein CopC |  |  |  |  |  |  |  |  |  |  |  |  |  |  |  |  |  |  |  |  |  |  |  |  |
| Copper resistance protein CopD |  |  |  |  |  |  |  |  |  |  |  |  |  |  |  |  |  |  |  |  |  |  |  |  |
| Copper resistance protein D |  |  |  |  |  |  |  |  |  |  |  |  |  |  |  |  |  |  |  |  |  |  |  |  |
| Copper-sensing two-component system response CusR |  |  |  |  |  |  |  |  |  |  |  |  |  |  |  |  |  |  |  |  |  |  |  |  |
| Cu(I)-responsive transcriptional regulator |  |  |  |  |  |  |  |  |  |  |  |  |  |  |  |  |  |  |  |  |  |  |  |  |
| Cytochrome c heme lyase subunit CcmF |  |  |  |  |  |  |  |  |  |  |  |  |  |  |  |  |  |  |  |  |  |  |  |  |
| Cytoplasmic copper homeostasis protein CutC |  |  |  |  |  |  |  |  |  |  |  |  |  |  |  |  |  |  |  |  |  |  |  |  |
| DNA-binding heavy metal response regulator |  |  |  |  |  |  |  |  |  |  |  |  |  |  |  |  |  |  |  |  |  |  |  |  |
| Heavy metal resistance transcriptional regulator HmrR |  |  |  |  |  |  |  |  |  |  |  |  |  |  |  |  |  |  |  |  |  |  |  |  |
| Heavy metal RND efflux outer memb. protein, CzcC |  |  |  |  |  |  |  |  |  |  |  |  |  |  |  |  |  |  |  |  |  |  |  |  |
| Heavy metal sensor histidine kinase |  |  |  |  |  |  |  |  |  |  |  |  |  |  |  |  |  |  |  |  |  |  |  |  |
| internalin, putative |  |  |  |  |  |  |  |  |  |  |  |  |  |  |  |  |  |  |  |  |  |  |  |  |
| Mercuric ion reductase (EC 1.16.1.1) |  |  |  |  |  |  |  |  |  |  |  |  |  |  |  |  |  |  |  |  |  |  |  |  |
| Mercuric resistance operon coregulator |  |  |  |  |  |  |  |  |  |  |  |  |  |  |  |  |  |  |  |  |  |  |  |  |
| Mercuric resistance operon regulatory protein |  |  |  |  |  |  |  |  |  |  |  |  |  |  |  |  |  |  |  |  |  |  |  |  |
| Mercuric transport protein, MerC |  |  |  |  |  |  |  |  |  |  |  |  |  |  |  |  |  |  |  |  |  |  |  |  |
| Mercuric transport protein, MerE |  |  |  |  |  |  |  |  |  |  |  |  |  |  |  |  |  |  |  |  |  |  |  |  |
| Mercuric transport protein, MerT |  |  |  |  |  |  |  |  |  |  |  |  |  |  |  |  |  |  |  |  |  |  |  |  |
| Periplasmic mercury(+2) binding protein |  |  |  |  |  |  |  |  |  |  |  |  |  |  |  |  |  |  |  |  |  |  |  |  |
| Organomercurial lyase (EC 4.99.1.2) |  |  |  |  |  |  |  |  |  |  |  |  |  |  |  |  |  |  |  |  |  |  |  |  |
| Transcription regulator protein of MDR efflux pump cluster |  |  |  |  |  |  |  |  |  |  |  |  |  |  |  |  |  |  |  |  |  |  |  |  |
| RND efflux system, memb. fusion protein CmeA |  |  |  |  |  |  |  |  |  |  |  |  |  |  |  |  |  |  |  |  |  |  |  |  |
| RND efflux system, outer memb. lipoprotein, NodT family |  |  |  |  |  |  |  |  |  |  |  |  |  |  |  |  |  |  |  |  |  |  |  |  |
| Transcriptional regulator NfxB |  |  |  |  |  |  |  |  |  |  |  |  |  |  |  |  |  |  |  |  |  |  |  |  |
| Two-component response regulator CreB |  |  |  |  |  |  |  |  |  |  |  |  |  |  |  |  |  |  |  |  |  |  |  |  |
| Two-component response regulator CreC |  |  |  |  |  |  |  |  |  |  |  |  |  |  |  |  |  |  |  |  |  |  |  |  |
| Inner membrane protein CreD |  |  |  |  |  |  |  |  |  |  |  |  |  |  |  |  |  |  |  |  |  |  |  |  |

* Genes present in all isolates are not included in the table.

#Strains with complete genomes available.

**Table S3. Matrix of presence/absence of virulence factors**

|  |  | Clinical strains | | | | | | | | | | | | Environmental strains | | | | | | | | | | | |
| --- | --- | --- | --- | --- | --- | --- | --- | --- | --- | --- | --- | --- | --- | --- | --- | --- | --- | --- | --- | --- | --- | --- | --- | --- | --- |
| **Gene** | **Gene ID** | **K279a*** | **D457*** | **E861** | **D388** | **E539** | **C357** | **E824** | **E729** | **E999** | **G51** | **E301** | **E759** | **R551-3*** | **JV3*** | **EA23** | **EP13** | **NS26** | **EP20** | **EA1** | **EA22** | **EA63** | **EA21** | **PS5** | **EP5** |
| Phospholipase D_3 | GI:504458309 (D457) |  |  |  |  |  |  |  |  |  |  |  |  |  |  |  |  |  |  |  |  |  |  |  |  |
| Metalloprotease | GI:754361759 (D457) |  |  |  |  |  |  |  |  |  |  |  |  |  |  |  |  |  |  |  |  |  |  |  |  |
| Serine-protease | GI:504458833 (D457) |  |  |  |  |  |  |  |  |  |  |  |  |  |  |  |  |  |  |  |  |  |  |  |  |
| Serine-protease | GI:754361938 (D457) |  |  |  |  |  |  |  |  |  |  |  |  |  |  |  |  |  |  |  |  |  |  |  |  |
| Serine-protease | GI:754361966 (D457) |  |  |  |  |  |  |  |  |  |  |  |  |  |  |  |  |  |  |  |  |  |  |  |  |
| Metalloprotease | GI:504459393 (D457) |  |  |  |  |  |  |  |  |  |  |  |  |  |  |  |  |  |  |  |  |  |  |  |  |
| secretion periplasmic adaptor | GI:491555445 (D457) |  |  |  |  |  |  |  |  |  |  |  |  |  |  |  |  |  |  |  |  |  |  |  |  |
| Phospholipase D_1 | GI:754362212 (D457) |  |  |  |  |  |  |  |  |  |  |  |  |  |  |  |  |  |  |  |  |  |  |  |  |
| T5SS autotransporter hemagglutinin_2 | GI:754362361 (D457) |  |  |  |  |  |  |  |  |  |  |  |  |  |  |  |  |  |  |  |  |  |  |  |  |
| Siderophore enterobactin synthetase_3 | GI:504460319 (D457) |  |  |  |  |  |  |  |  |  |  |  |  |  |  |  |  |  |  |  |  |  |  |  |  |
| DNase | GI:504460615 (D457) |  |  |  |  |  |  |  |  |  |  |  |  |  |  |  |  |  |  |  |  |  |  |  |  |
| Phospholipase A | GI:504460620 (D457) |  |  |  |  |  |  |  |  |  |  |  |  |  |  |  |  |  |  |  |  |  |  |  |  |
| Serine-protease | GI:754362539 (D457) |  |  |  |  |  |  |  |  |  |  |  |  |  |  |  |  |  |  |  |  |  |  |  |  |
| Hemolysin III | GI:504460964 (D457) |  |  |  |  |  |  |  |  |  |  |  |  |  |  |  |  |  |  |  |  |  |  |  |  |
| Autotransporter lipase/esterase | GI:504461110 (D457) |  |  |  |  |  |  |  |  |  |  |  |  |  |  |  |  |  |  |  |  |  |  |  |  |
| Protease IV | GI:754362756 (D457) |  |  |  |  |  |  |  |  |  |  |  |  |  |  |  |  |  |  |  |  |  |  |  |  |
| Serine-protease | GI:504461623 (D457) |  |  |  |  |  |  |  |  |  |  |  |  |  |  |  |  |  |  |  |  |  |  |  |  |
| Serine-protease | GI:504461644 (D457) |  |  |  |  |  |  |  |  |  |  |  |  |  |  |  |  |  |  |  |  |  |  |  |  |
| Non fimbrial adhesin | GI:754362796 (D457) |  |  |  |  |  |  |  |  |  |  |  |  |  |  |  |  |  |  |  |  |  |  |  |  |
| Phospholipase D_2 | GI:504461767 (D457) |  |  |  |  |  |  |  |  |  |  |  |  |  |  |  |  |  |  |  |  |  |  |  |  |
| Phospholipase B_1 | GI:696384648 (D457) |  |  |  |  |  |  |  |  |  |  |  |  |  |  |  |  |  |  |  |  |  |  |  |  |
| Metalloprotease | GI:754361869 (D457) |  |  |  |  |  |  |  |  |  |  |  |  |  |  |  |  |  |  |  |  |  |  |  |  |
| Phospholipase C | GI:504459627 (D457) |  |  |  |  |  |  |  |  |  |  |  |  |  |  |  |  |  |  |  |  |  |  |  |  |
| Siderophore enterobactin synthetase_1 | GI:504460314 (D457) |  |  |  |  |  |  |  |  |  |  |  |  |  |  |  |  |  |  |  |  |  |  |  |  |
| Siderophore enterobactin synthetase_2 | GI:504460315 (D457) |  |  |  |  |  |  |  |  |  |  |  |  |  |  |  |  |  |  |  |  |  |  |  |  |
| Hemoglobin binding protein | GI:504460839 (D457) |  |  |  |  |  |  |  |  |  |  |  |  |  |  |  |  |  |  |  |  |  |  |  |  |
| Hemoglobin binding protein | GI:504461648 (D457) |  |  |  |  |  |  |  |  |  |  |  |  |  |  |  |  |  |  |  |  |  |  |  |  |
| Serine-protease | GI:504458550 (D457) |  |  |  |  |  |  |  |  |  |  |  |  |  |  |  |  |  |  |  |  |  |  |  |  |
| T5SS autotransporter hemagglutinin_1 | GI:504460261 (K279a) |  |  |  |  |  |  |  |  |  |  |  |  |  |  |  |  |  |  |  |  |  |  |  |  |
| Filamentous hemagglutinin | GI:765003484 (K279a) |  |  |  |  |  |  |  |  |  |  |  |  |  |  |  |  |  |  |  |  |  |  |  |  |
| Hemagglutinin like adhesin | GI:765004612 (K279a) |  |  |  |  |  |  |  |  |  |  |  |  |  |  |  |  |  |  |  |  |  |  |  |  |
| Putative Lipase | GI:501455617 (K279a) |  |  |  |  |  |  |  |  |  |  |  |  |  |  |  |  |  |  |  |  |  |  |  |  |
| Filamentous hemaglutinin | GI:493444609 (RA8) |  |  |  |  |  |  |  |  |  |  |  |  |  |  |  |  |  |  |  |  |  |  |  |  |
| Serine-protease | GI:493412491 (RA8) |  |  |  |  |  |  |  |  |  |  |  |  |  |  |  |  |  |  |  |  |  |  |  |  |
